# Development of a cooperative two-factor adaptive-evolution method to enhance lipid production and prevent lipid peroxidation in *Schizochytrium* sp.

**DOI:** 10.1186/s13068-018-1065-4

**Published:** 2018-03-14

**Authors:** Xiao-Man Sun, Lu-Jing Ren, Zhi-Qian Bi, Xiao-Jun Ji, Quan-Yu Zhao, Ling Jiang, He Huang

**Affiliations:** 1 College of Biotechnology and Pharmaceutical Engineering, Nanjing Tech University, No. 30 South Puzhu Road, Nanjing, 211816 People’s Republic of China; 2 School of Pharmaceutical Sciences, Nanjing Tech University, No. 30 South Puzhu Road, Nanjing, 211816 People’s Republic of China; 3 State Key Laboratory of Materials-Oriented Chemical Engineering, Nanjing Tech University, No. 5 Xinmofan Road, Nanjing, 210009 People’s Republic of China; 4 Jiangsu National Synergetic Innovation Center for Advanced Materials (SICAM), Nanjing Tech University, No. 5 Xinmofan Road, Nanjing, 210009 People’s Republic of China

**Keywords:** *Schizochytrium* sp., Docosahexaenoic acid, Adaptive evolution, Lipid peroxidation, Antioxidant enzyme

## Abstract

**Background:**

*Schizochytrium* sp. is a marine microalga with great potential as a promising sustainable source of lipids rich in docosahexaenoic acid (DHA). This organism’s lipid accumulation machinery can be induced by various stress conditions, but this stress induction usually comes at the expense of lower biomass in industrial fermentations. Moreover, oxidative damage induced by various environmental stresses can result in the peroxidation of lipids, and especially polyunsaturated fatty acids, which causes unstable DHA production, but is often ignored in fermentation processes. Therefore, it is urgent to develop new production strains that not only have a high DHA production capacity, but also possess strong antioxidant defenses.

**Results:**

Adaptive laboratory evolution (ALE) is an effective method for the development of beneficial phenotypes in industrial microorganisms. Here, a novel cooperative two-factor ALE strategy based on concomitant low temperature and high salinity was applied to improve the production capacity of *Schizochytrium* sp. Low-temperature conditions were used to improve the DHA content, and high salinity was applied to stimulate lipid accumulation and enhance the antioxidative defense systems of *Schizochytrium* sp. After 30 adaptation cycles, a maximal cell dry weight of 126.4 g/L and DHA yield of 38.12 g/L were obtained in the endpoint strain ALE-TF30, which was 27.42 and 57.52% higher than parental strain, respectively. Moreover, the fact that ALE-TF30 had the lowest concentrations of reactive oxygen species and malondialdehyde among all strains indicated that lipid peroxidation was greatly suppressed by the evolutionary process. Accordingly, the ALE-TF30 strain exhibited an overall increase of gene expression levels of antioxidant enzymes and polyketide synthases compared to the parental strain.

**Conclusion:**

This study provides important clues on how to overcome the negative effects of lipid peroxidation on DHA production in *Schizochytrium* sp. Taken together, the cooperative two-factor ALE process can not only increase the accumulation of lipids rich in DHA, but also prevent the loss of produced lipid caused by lipid peroxidation. The strategy proposed here may provide a new and alternative direction for the industrial cultivation of oil-producing microalgae.

**Electronic supplementary material:**

The online version of this article (10.1186/s13068-018-1065-4) contains supplementary material, which is available to authorized users.

## Background

Docosahexaenoic acid (DHA) plays important roles in alleviating cardiovascular diseases, hypertension, diabetes, and neuro-psychiatric disorders [1]. Due to these positive effects on human health, DHA is of considerable interest as a food additive and pharmaceutical target. Marine fish are traditionally known as a natural source of DHA. However, the limitations of this source, such as seasonal availability, location, the ecological impact of fishing, and the resulting environmental pollution, make it difficult to fulfill the nutritional requirements of human beings [2]. In fact, the accumulation of DHA in marine fish is largely attributed to their consumption of microalgae. Hence, microalgae have a significant potential as sources of omega-3 polyunsaturated fatty acids (PUFA). The marine microalga *Schizochytrium* sp. is famous for producing significant amounts of DHA, in conjunction with a fast growth rate and high productivity [3].

In general, lipids act as an energy-rich carbon storage battery, and are synthesized by microalgae to retain their growth rate under extreme environmental conditions such as nitrogen limitation or high salinity [4]. Consequently, stress-based strategies are widely used as environmentally friendly approaches to induce lipid overproduction in cultured microorganisms [5]. However, stress-based strategies usually work at the expense of lower biomass. A general countermeasure to overcome this difficulty is to use a two-stage cultivation strategy, dedicating the first stage with optimum growth conditions to the maximization of biomass production, while reserving the second stage for the accumulation of lipids under stress conditions. However, such multi-stage cultivation strategies consume more energy, and the exact time-point of switching the conditions for fermentation control is difficult to determine accurately, leading to high costs and production instability [6]. Consequently, stable mutants with improved tolerance to the process conditions should be developed to enhance lipid yield.

Moreover, formation of reactive oxygen species (ROS) is an inevitable aspect of cellular responses to stress-based strategies. It is well established that ROS can react instantaneously and non-specifically with essential biological molecules, and lead to the alteration of cellular functions by inducing DNA damage and mutations, protein oxidation, or peroxidation of lipids [7]. The lipids of microalgae contain a high percentage of PUFAs and are consequently susceptible to peroxidation because the unsaturated double bonds in the PUFAs are more prone to oxidation [8]. Lipid peroxidation not only leads to the accumulation of high levels of ROS, but also causes a loss of the produced lipids, which unfortunately must often be ignored in fermentation processes. Cells experience oxidative stress when the amount of ROS exceeds the capacity of the antioxidant defense systems to detoxify them [9]. Hence, it is urgent to obtain a strain of *Schizochytrium* sp. with combined characteristics of high production and strong antioxidant defense systems to prevent lipid peroxidation. There are several approaches available to achieve this end, mostly involving genetic engineering techniques. However, the use of genetically modified strains in the food industry is not permitted in many parts of the world, and consumer acceptance of such technologies remains a contentious issue [10]. To overcome these bottlenecks, an approach such as adaptive laboratory evolution (ALE) could be beneficial.

ALE has been widely used to develop novel biological and phenotypic functions, and also for the improvement of strains obtained through synthetic biology, including important model organisms such as *Saccharomyces cerevisiae* and *Escherichia coli* [11]. Briefly, ALE subjects microbes to repeated or continuous cultivation under stress conditions for many generations to enrich favorable genetic changes or achieve better inhibitor tolerance [12]. In recent years, many successful ALE experiments were applied to improve the production of lipids and other high-value-added products in microalgae. For example, a highly glucose-tolerant strain of *Crypthecodinium cohnii* and a highly glycerol-tolerant strain of *Rhodococcus opacus* were obtained by ALE to relieve substrate inhibition, which exhibited improved growth and lipid accumulation at high glucose and glycerol concentrations, respectively [13, 14]. In addition to nutrient stress, various environmental stress factors such as high oxygen [15], high temperature [16], or high salt concentrations [17] were applied in ALE to improve microalgal performance. Thus, ALE has been gradually introduced as a novel, powerful tool to improve the strain properties of microalgae. The increase of the PUFA percentage at low culture temperatures is a common trend reported for microalgae. For example, a temperature decrease yielded two- to threefold increase of EPA and DHA contents in several mesophilic species [18, 19]. In addition, many recent studies have found significant effects of salinity on the concentrations of important antioxidants in microalgae. In *Dunaliella tertiolecta*, hypersaline stress can induce a massive increase of the hydrogen peroxide scavenging capacity [20]. We therefore hypothesized that a combined low-temperature/high-salinity adaption strategy could be applied to simultaneously improve DHA synthesis and antioxidant defense systems of *Schizochytrium* sp., which would arguably lead to better industrial strains.

Very few studies to date have focused on the effects of oxidative damage on the fermentation performance of microalgae. In addition, we are not aware of any studies devoted to the development of a high antioxidant capacity in this organism. In this study, we successfully established a cooperative two-factor ALE strategy, including low temperature and high salinity, to simultaneously improve the growth and DHA production of *Schizochytrium* sp. The best evolved clones exhibited significantly increased product formation and high antioxidant capacity. ROS, T-AOC, and MDA were used to compare the antioxidant defense systems of the endpoint strain and starting strain. In addition, seven genes associated with antioxidant enzymes and fatty acid biosynthesis were analyzed by qRT-PCR. This study thus provides a new ALE method that can improve multiple factors of strain productivity, which also might be used in other oil-producing microalgae.

## Results

### Changes of the growth properties of *Schizochytrium* sp. during ALE

**Fig. 1:**
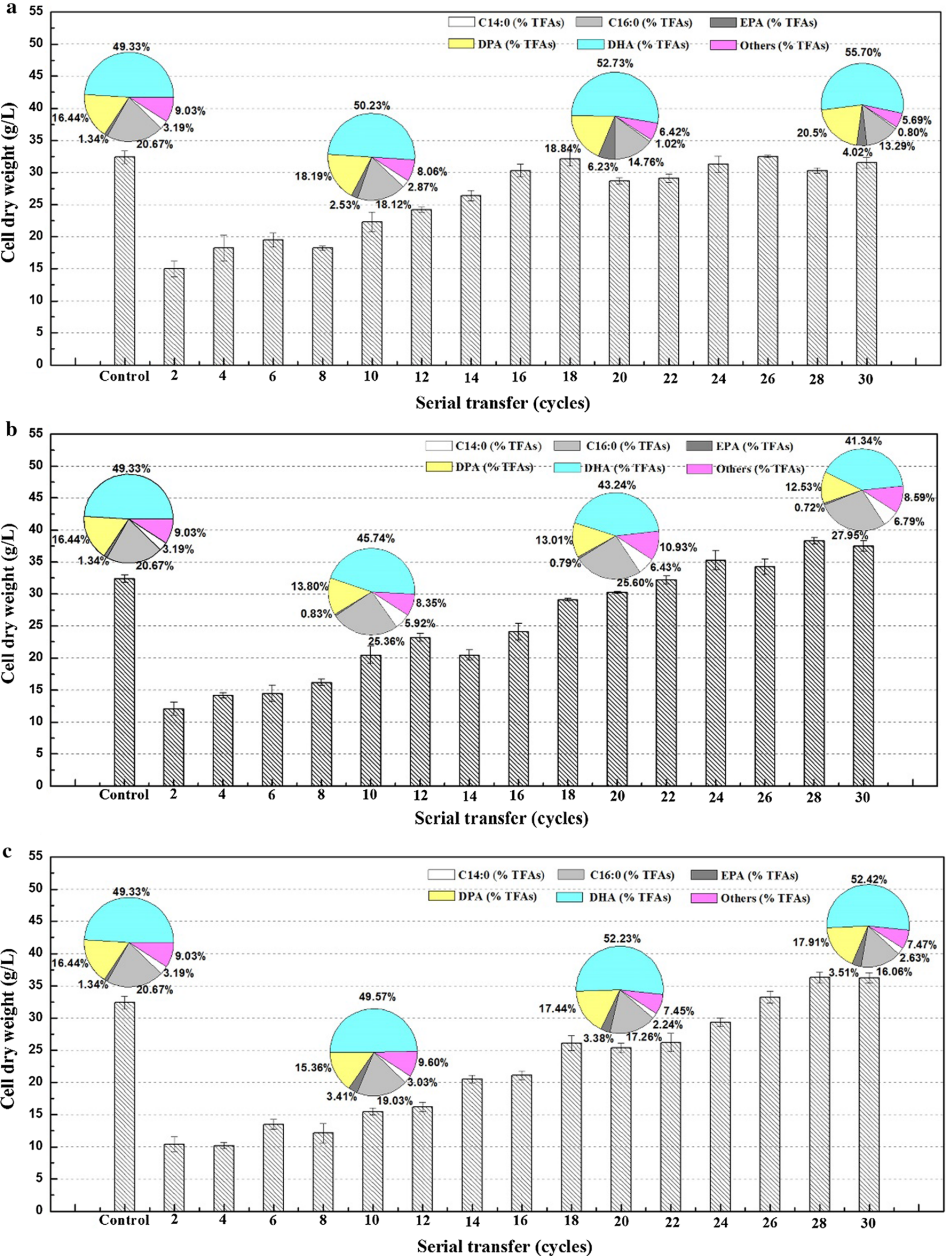
Cell dry weight (CDW) and fatty acid content (as % of TFAs) of different evolved strains during adaptive laboratory evolution. Values and error bars represent the means and the standard deviations of triplicate experiments. **a** Low-temperature ALE, **b** high-salinity ALE, **c** cooperative two-factor ALE

We analyzed samples collected each 2 cycles to monitor the cell dry weight (CDW) and analyzed the fatty acid profiles each 10 cycles. These two indices were recognized as the first indicators of the success or failure of the adaption. As shown in Fig. 1a, at the end of the second cycle, the biomass concentration under low temperature and high salinity reached 15.0 and 12.1 g/L, which was 53.7 and 62.7% lower than that of the control, respectively. Furthermore, the combination of the two stress conditions resulted in a severe drop of CDW to 10.4 g/L, indicating that combined stresses hinder the growth of *Schizochytrium* sp. more than the individual stress factors. With the progress of the ALE process, a clear increasing trend of biomass became visible in spite of considerable fluctuations. The strains undergoing low-temperature ALE took 18 cycles to recover the original growth rate under optimal conditions, at which point the CDW was 32.4 g/L—the same as that of the control. By contrast, the recovery of the original CDW took 22 cycles in the high-salinity ALE, indicating that *Schizochytrium* sp. requires a greater length of time to obtain a stable improved phenotype under high-salinity stress. Similarly, strains in the cooperative two-factor ALE took the longest to adapt, reaching the CDW of the control only after 28 cycles. In spite of the difficulty of adaptation, ALE-LT30, ALE-HS30, and ALE-TF30 all showed increased growth compared to the parental strain.

Moreover, a significant difference was observed in the fatty acid composition between the staring strain and the evolved strains (Fig. 1). The saturated fatty acids (SFA) in *Schizochytrium* sp. are mainly composed of C14:0 and C16:0, whereas PUFAs mainly consist of EPA, docosapentaenoic acid (DPA), and DHA. As shown in Fig. 1, the strains’ biosynthesis capacity for SFA and PUFA followed opposite trajectories under all adaptation conditions. With increasing adaptation time under low temperature, the PUFA percentage in TFAs increased over time from 67.11% at the beginning to 80.22% at 30 cycles. Importantly, EPA accumulation increased significantly after 20 cycles of low-temperature ALE, resulting in an EPA content of 6.23% of TFAs, which was 4.6-fold higher than that of the starting strain. Moreover, the DHA percentage showed a peak value 55.70% after 30 cycles, which was 12.91% higher than that of the starting strain. However, an opposite trend was observed in the high-salinity ALE, whereby SFA increased and PUFA decreased over time. Unfortunately, the PUFA percentage continued to decrease and finally reached 54.59% in the endpoint strain ALE-HS30, which was 18.65% lower than that of the starting strain. Cooperative two-factor ALE also enhanced the PUFA percentage during the entire time course, although the increment was lower than in the low-temperature ALE experiment, reflecting the strong influence of low-temperature stress on fatty acid composition.

### Isolation and comparison of the most promising evolved strains

In an attempt to evaluate the effects of the three ALE methods and isolate the most promising evolved strains, we investigated the changes of the fermentation characteristics between the starting strain and the endpoint strains ALE-LT30, ALE-HS30, and ALE-TF30, cultured under the same conditions at 30 °C in normal fermentation medium. All of the strains were able to complete the fermentation, although the duration of the fermentation differed between the evolved strains (Table 1). Two evolved strains, ALE-SH30 and ALE-TF30, consumed all of the sugar in less than 45 h, the same time as the ancestral strain, whereas ALE-LT30 took an additional 8 h. Finally, the observation that the CDW of ALE-LT30 was the lowest further reflected the fact that the low-temperature adaptation actually hindered the cell growth of *Schizochytrium* sp. By contrast, the biomass concentrations of the evolved strains ALE-HS30 and ALE-TF30 were increased to 49.85 and 47.23 g/L, which were 23.39 and 16.91% higher than in the parental strain, respectively (Table 1). Moreover, the strains adapted to high salinity, including ALE-HS30 and ALE-TF30, exhibited positive changes of lipid accumulation. In these cultures, the maximum lipid production achieved by ALE-HS30 was 20.45 g/L, representing 41.02% of CDW after 42-h cultivation, which was 51.48% higher than that of the parental strain.

**Table 1:** Fermentation characteristics of different evolved strains at the end of fermentation

Parameter	Strain			
	Parental strain	ALE-LT30	ALE-HS30	ALE-TF30
CDW (g/L)	40.4 ± 0.8	36.10 ± 1.1	49.85 ± 1.3	47.23 ± 0.9
TL concentration (g/L)	13.5 ± 0.6	10.13 ± 0.8	20.45 ± 0.5	17.76 ± 1.2
TLs in CDW (w/w, %)	33.41	28.06	41.02	37.60
Fermentation time (h)	42	50	42	45
DHA yield (g/L)	6.58 ± 0.4	5.49 ± 0.5	9.04 ± 0.8	9.49 ± 0.5
PUFA/SFA	2.96	5.20	2.06	4.56

Data represent the mean values and standard deviations of three replicates for each measurement

**Fig. 2:**
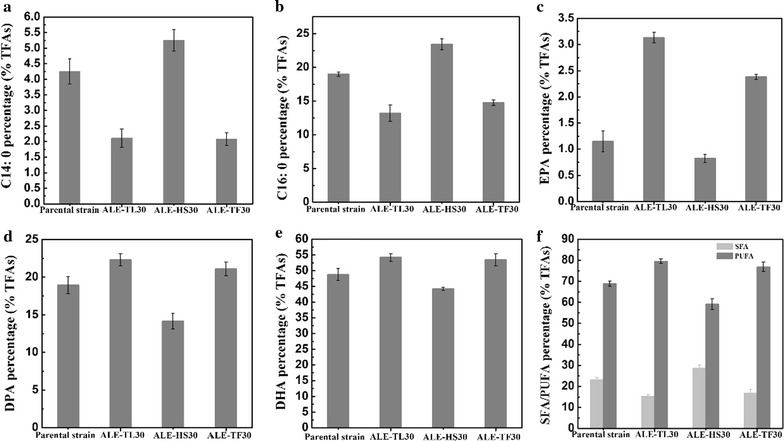
Fatty acid content (%TFAs) of the starting strain and evolved strains (ALE-LT30, ALE-HS30 and ALE-TF30) at the end of fermentation. **a** C14:0 percentage (% TFAs). **b** C14:0 percentage (% TFAs). **c** EPA percentage (% TFAs), **d** DPA percentage (% TFAs)SFA, **e** DHA percentage (% TFAs) content, **f** SFA represents the summation of C14:0 and C16:0. PUFA represents the summation of EPA, DPA and DHA. Values and error bars represent the means and the standard deviations of triplicate experiments

Interestingly, ALE exhibited significant effects not only on the total lipids, but also on the fatty acid composition (Fig. 2). Similarly, the PUFA percentage of the ALE-LT30 strain increased to 79.62% and the SFA percentage decreased to 15.32%, compared with 68.82 and 23.23% in the staring strain, respectively. Thus, the low-temperature ALE strains still kept a significant advantage in EPA accumulation. Conversely, a higher SFA percentage (28.68%) and lower PUFA percentage (59.15%) were observed with the evolved strain ALE-HS30, which indicated that high-salinity adaptation was advantageous for improving cell growth and lipid production, albeit at the expense of the PUFA yield. Interestingly, ALE-TF30 combined the advantages of higher PUFA accumulation of the low-temperature ALE and higher cell growth and lipid accumulation of the high-salinity ALE. At the end of fermentation, the lipid production and DHA percentage of ALE-TF30 reached 17.76 g/L and 53.43%, respectively, thus giving a maximum DHA yield of 9.49 g/L, which was 44.22% higher than that of the parental strain. The ALE-TF30 strain therefore was the most promising evolved strain in terms of fermentation performance and DHA production. Moreover, the results of a passage experiment showed that endpoint strain ALE-TF30 had stable genetics in terms of producing a higher cell dry weight and DHA percentage in TFAs (Additional file 1: Table S1).

**Fig. 3:**
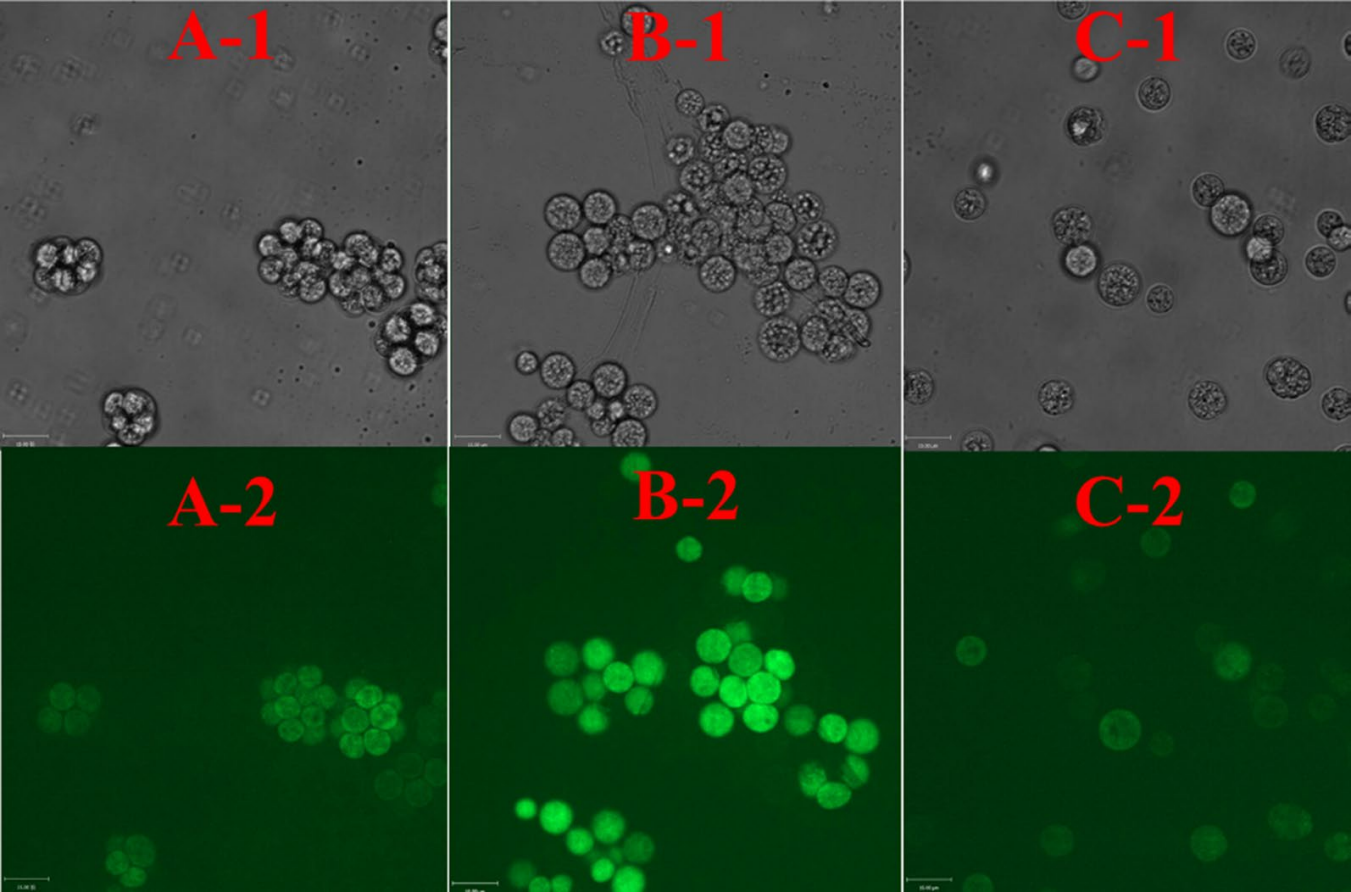
Determination of cell morphology and ROS levels using DCFH-DA by confocal laser microscopy. **A** The parental strain cultured in normal medium for 1 cycle. **B** The parental strain cultured under low-temperature and high-salinity conditions for 1 cycle. **C** The evolved strain ALE-TF30 cultured in normal medium for 1 cycle

To further explore the stress status of the parental strain and the endpoint strain under the tested experimental conditions, the morphology and oxidative stress of the cells were investigated by confocal laser microscopy. Intracellular ROS react with the DCFH-DA probe to produce an oxidized fluorophore exhibiting green fluorescence at an excitation wavelength of 485 nm with an emission wavelength of 530 nm, whereby the fluorescence intensity reflects the degree of oxidative damage in the cells. Figure 3B shows the data from the parental strain cultured under combined low-temperature/high-salinity conditions for 1 cycle. At this point, the cells that were not adapted to environmental stress showed a severely damaged, clustered appearance (Fig. 3B-1), similar to what was observed in *Chlamydomonas reinhardtii* [21]. Moreover, strong green fluorescence indicated that the cells had suffered severe oxidative damage (Fig. 3B-2), much more than the parental strain (Fig. 3A-2). Interestingly, the ALE-TF30 strain showed signs of abnormal cell division stages, with individual cells found in isolation (Fig. 3C-1), as opposed to the multi-cell clusters of the parental strain (Fig. 3A-1). In addition, with the progress of the adaptive evolution, the cells gradually adapted to the low-temperature and high-salinity conditions, with lower green fluorescence in the strain ALE-TF30 (Fig. 3C-2) demonstrating its improved ROS-scavenging ability or higher antioxidant capacity.

### Characterization of the batch-fermentation behavior of the strains evolved via the cooperative two-factor ALE procedure

**Fig. 4:**
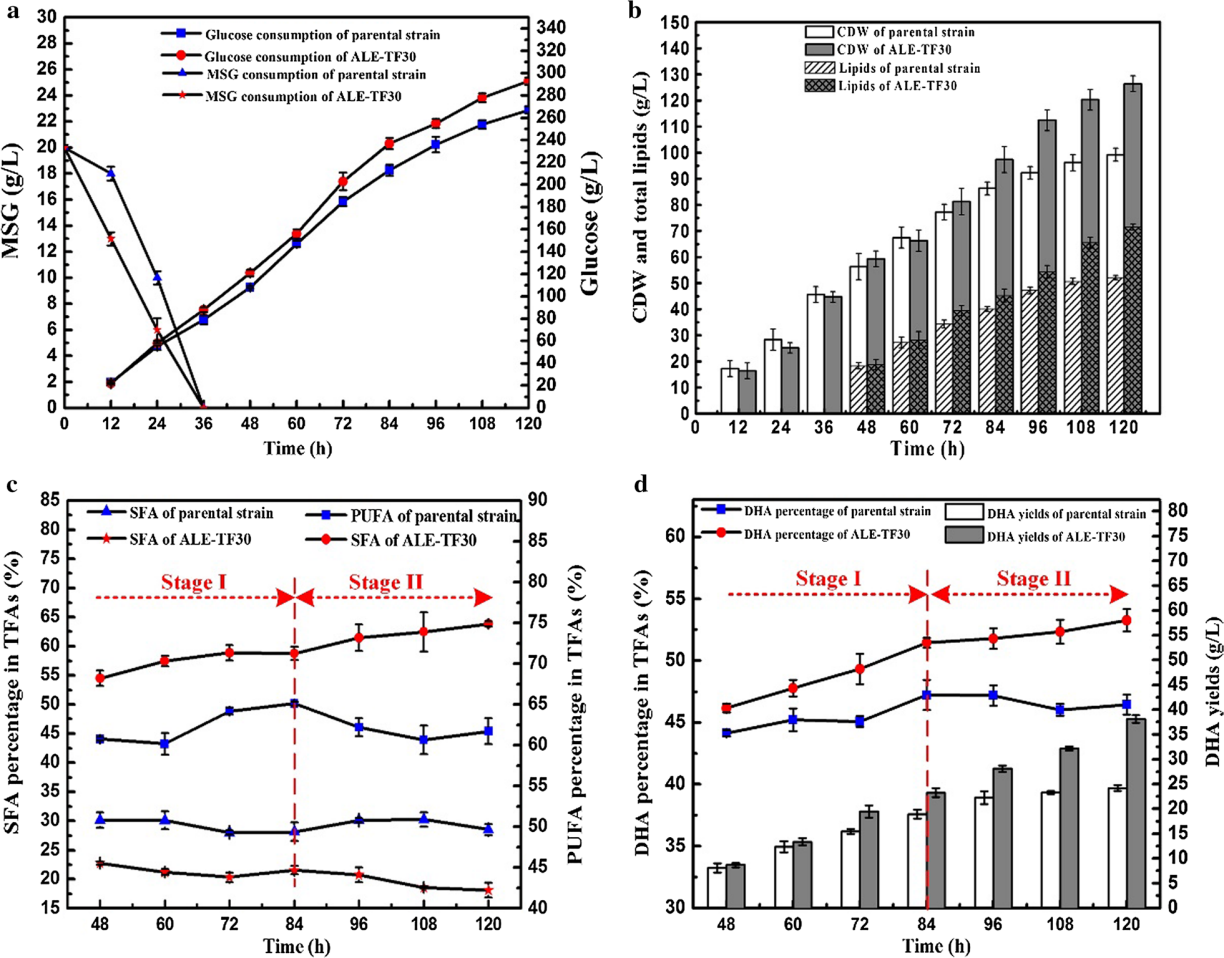
Comparison between the parental strain and the endpoint strain ALE-TF30 for substrate consumption (**a**), CDW and lipids (**b**), SFA and PUFA percentage in TFAs (**c**), as well as DHA percentage in TFAs and DHA yield (**d**) in a 5-L bioreactor. Values and error bars represent the means and the standard deviations of triplicate experiments

To further investigate and increase the ability of the evolved strain ALE-TF30 to produce DHA-rich lipids, we submitted it to conventional culture in a 5-L bioreactor. To better analyze the process of cell growth and lipid accumulation and understand the effects of lipid peroxidation on the production of PUFA, and especially DHA, the fermentation process was divided into two stages according to their PUFA accumulation characteristics. Stage I represents the process from the beginning until 84 h, which encompasses the phase of rapid cell growth and lipid accumulation. The PUFA percentage (as % of TFAs) increased gradually during this stage. Stage II was the oxidative damage phase (84–120 h) with severe lipid peroxidation and a decline of the PUFA percentage (as % of TFAs). As shown in Fig. 4a, b, in stage I, the evolved strain ALE-TF30 exhibited better growth and faster consumption of substrates than the parent strain, including the consumption of glucose and MSG. The strain’s superiority became more obvious in stage II, which yielded the largest CDW of 126.4 g/L by ALE-TF30 at 120 h, representing a 27.2% increase over the parental strain. After nitrogen exhaustion, the total lipid concentration increased sharply in both strains, from 18.32 and 18.77 g/L at 48 h to 40.12 and 45.23 g/L at 84 h, respectively. Nevertheless, the endpoint strain exhibited an overall better lipid productivity in stage II than the starting strain, e.g., 0.73 g/L/h from 84 to 120 h, which represented a 121% increase over the starting strain (Fig. 4b). The final lipid yield of the evolved strain ALE-TF30 was 71.56 g/L, which was 37.32% higher than that of the starting strain (Table 2). This implied that the strain ALE-TF30, i.e., the final product of the cooperative two-factor evolution possess, had a stronger antioxidant capacity, and thus fared better in the oxidative damage stage. In recent years, many efforts have been made to improve biomass and lipid accumulation by substrate optimization. For example, the biomass was increased to 88.6 g/L by a MSG-feeding strategy in *Schizochytrium* sp. LU310 [22], which was favorable for the later lipid accumulation stage. Moreover, when a combination of yeast extract and MSG was used, 55.83 g/L of biomass and a lipid productivity of 0.203 g/L/h were obtained in *Schizochytrium* sp. ATCC 20888 [23].

**Table 2:** Fermentation parameters of the starting strain and endpoint strains at 120 h in a 5-L bioreactor

	Starting strain	Endpoint strain (ALE-TF30)	Increase (%)
CDW (g/L)	99.2 ± 2.5	126.4 ± 3.1	27.42
Lipid yield (g/L)	52.11 ± 0.8	71.56 ± 1.2	37.32
DHA percentage (% TFAs)	46.44 ± 0.8	53.27 ± 0.9	14.71
DHA yield (g/L)	24.2	38.12	57.52
DHA productively (mg/L/h)	201.67	317.67	57.52

Data represent the mean values and standard deviations of three replicates for each measurement

Similarly, the PUFA and DHA percentages of the parental strain (as % of TFAs) reached a maximum of 65.11% and 47.23% at 84 h, respectively, followed by a decline in stage II (Fig. 4c). By contrast, the strain ALE-TF30 showed a continuous increase of the PUFA and DHA percentage during the entire fermentation process, with respective maximal values of 74.88 and 53.27% obtained at 120 h. Hence, the highest DHA yield of 38.12 g/L was obtained at 120 h in the endpoint strain, which was 57.52% higher than that of the parental strain (Fig. 4d, Table 2). These results clearly indicate that cooperative two-factor ALE can effectively promote the production of lipids and DHA, especially at stage II. In addition to ALE, mutagenesis is widely used as a simple and effective breeding strategy to produce mutant strains for DHA hyperproduction. The mutant strain *Schizochytrium* sp. S1, which was obtained via by low-energy ionizing radiation treatment, showed a higher DHA percentage of 46.2% [24]. Moreover, in our previous study, a DHA percentage of up to 56.22% was achieved using *N*-methyl-N0-nitro-*N*-nitrosoguanidine induction coupled with ultraviolet mutagenesis [25].

### Evaluation of lipid peroxidation

**Fig. 5:**
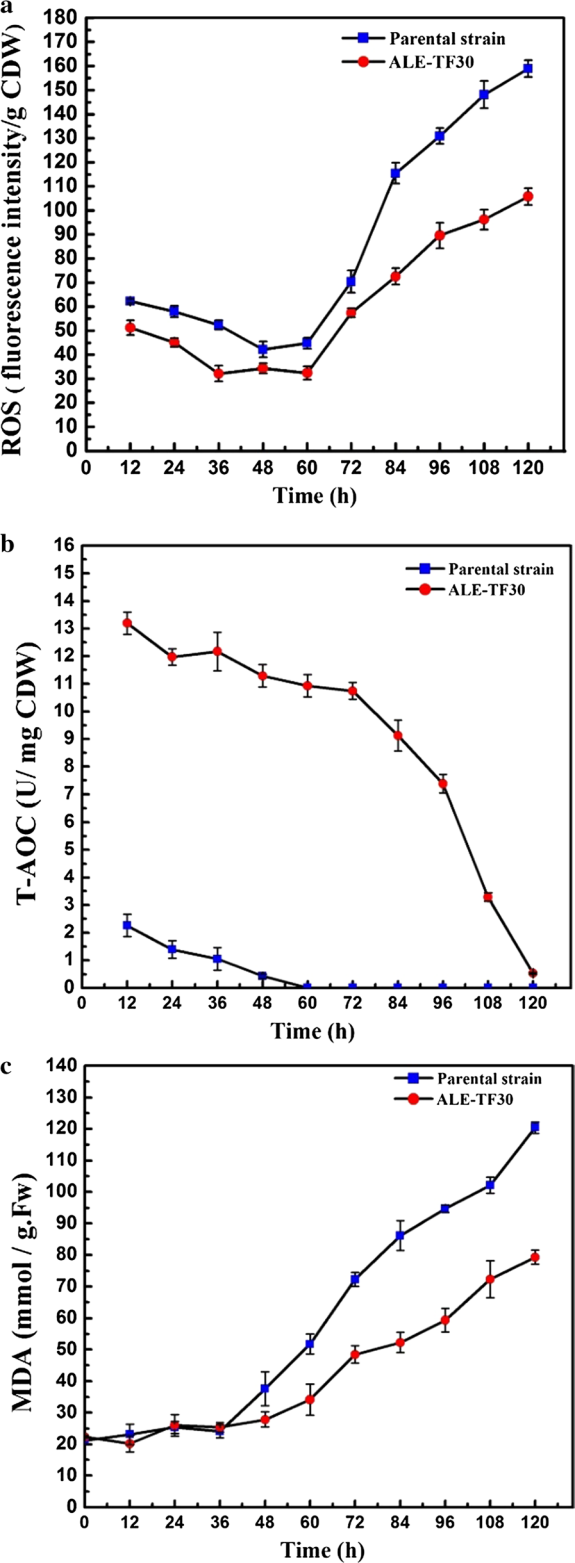
Comparison between the parental strain and the endpoint strain ALE-TF30 for ROS (**a**), T-AOC (**b**), and MDA (**c**) in a 5-L bioreactor. Values and error bars represent the means and the standard deviations of triplicate experiments

In order to study the connection between lipid accumulation and oxidative damage, the intracellular amounts of ROS and T-AOC were determined (Fig. 5a, b). Moreover, lipid peroxidation is another commonly used stress marker, which can be measured by determining the malondialdehyde (MDA) content in the cells (Fig. 5c). In both strains, the ROS levels decreased before 48 h, and subsequently dramatically increased from 60 h until the end of fermentation. Compared with the starting strain, a significant drop in the ROS levels occurred in ALE-TF30, especially after 84 h, leading to a minimum ROS level of 105.7 (fluorescence intensity/g CDW) at 120 h, which was 33.50% lower than that of the parental strain. Overall, the T-AOC of all cultures decreased with increasing fermentation time. The parental strain maintained a non-detectable level from 60 h until the end of fermentation, which demonstrates that wild-type *Schizochytrium* sp. has a serious deficiency of T-AOC, whereby the T-AOC values of the evolved strain were significantly higher and maintained an overall stronger antioxidant capacity during the entire fermentation period. MDA is a major indicator of damage to lipids and increased lipid peroxidation, which indicates severe oxidative stress. The MDA content was increased in both strains after 36 h, but the increase observed in ALE-TF30 was significantly lower than that of the parental strain. These results highlight the stronger antioxidative capacity of the evolved strain, which resulted in a much better defense against lipid peroxidation.

### Verification of the expression levels of key enzymes by RT-qPCR

**Fig. 6:**
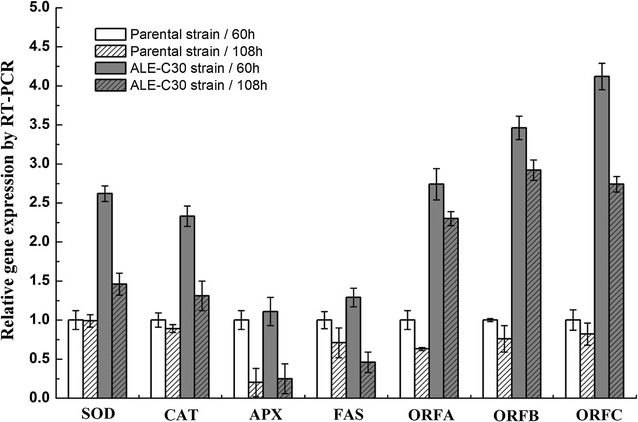
Real-time quantitative PCR results for the SOD, CAT, APX, FAS, ORFA, ORFB, and ORFC genes from the parental strain and endpoint strain ALE-TF30. Values and error bars represent the means and the standard deviations of triplicate experiments

To validate the metabolic observations, the three key genes associated with oxidative stress (SOD, CAT, and APX), and four key genes of fatty acid biosynthesis (FAS, ORFA, ORFB, ORFC) were analyzed by qRT-PCR at 60 and 108 h (Fig. 6). In microalgae, a number of biological defense mechanisms have evolved to neutralize ROS, including the action of antioxidant enzymes such as superoxide dismutase (SOD), catalase (CAT), and ascorbate peroxidase (APX) [26]. The steps involving the conversion of the exceedingly toxic superoxide anion (O_2_^−^) into merely toxic hydrogen peroxide (H_2_O_2_) by SOD and the subsequent disproportionation of H_2_O_2_ into innocuous O_2_ and H_2_O by CAT are very important [27, 28]. In addition, H_2_O_2_ can also be scavenged by APX using ascorbate as a sacrificial substrate [29]. Compared to the parental strain, an overall increase of the gene expression levels of SOD (2.62-fold higher), CAT (2.33-fold higher), and APX (11% higher) was seen in the ALE-TF30 strain at 60 h. However, as the culture time increased to 108 h, the expression of SOD, CAT, and APX was downregulated in both strains.

In *Schizochytrium* sp., FAS is a crucial contributor to SFA biosynthesis, and ORFA, ORFB, and ORFC, encoding polyketide synthases, are responsible for the synthesis of LC-PUFAs [30]. As shown in Fig. 6, cooperative two-factor ALE led to a slight increase of FAS gene expression. Interestingly, the ALE-TF30 strain displayed a much higher increase of the gene expression of the PUFA synthase subunits ORFA, ORFB, and ORFC, which were approximately 2.74-, 3.46-, and 4.12-fold higher than in the parental strain at 60 h, respectively. Similarity, the expression of these three genes was downregulated after entering the oxidative damage stage. Nevertheless, the evolved strain maintained significantly stronger expression of ORFA, ORFB, and ORFC (2.30-, 2.92-, 2.74-fold higher than in the parental strain) at 120 h, indicating that the metabolic fluxes towards the FAS and PKS pathways were changed by the evolutionary process.

## Discussion

In this study, a novel cooperative two-factor adaptive laboratory evolution (ALE) protocol was developed to create a stable improved strain that efficiently produces PUFA-rich lipids. In microalgae, temperature stress generally has a greater effect on the lipid profile than on overall lipid production. For instance, the DHA fraction in the TFA of *Aurantiochytrium mangrovei* increased from 29 to 42% in the late lipid accumulation phase when the temperature was decreased from 30 to 12 °C [31]. However, high salinity can stimulate lipid accumulation in microalgae [32], which can be attributed to osmotic stress that may be analogous to nutrient stress regarding the cellular responses [5]. Moreover, Van et al. reported that NaCl was the most efficient inducer of antioxidant enzymes in microalgae [33]. Unfortunately, the higher accumulation of lipids or PUFA induced via low temperature and high salinity is usually achieved at the expense of lower biomass. Hence, a cooperative two-factor ALE strategy including both low temperature and high salinity was proposed to evolve more robust and promising mutants. On one hand, low-temperature conditions were used to improve the PUFA content. On the other hand, high-salinity conditions were applied to stimulate lipid accumulation and enhance the antioxidative defense systems of *Schizochytrium* sp. Cooperative two-factor ALE therefore not only increased the accumulation of PUFA-rich lipids, but also prevented the loss of lipids caused by peroxidation.

Under low-temperature conditions, more PUFAs are synthesized and incorporated into the membrane to maintain its fluidity and permeability [34], and the enhancement of PUFA production in cells under temperature-stress conditions has been studied early on by Béchet et al. [35]. A recent proteomics study indicated that cold stress inhibits the cellular supply of energy from glycolysis and the TCA cycle [36], which might be the key reason for the low CDW observed in the low-temperature ALE experiment. However, the increase of EPA percentage was more remarkable than that of DPA or DHA in low-temperature ALE (Figs. 1a, 2c). Many studies suggested that EPA is not required for bulk bilayer fluidity but plays a beneficial role in membrane organization at low temperatures, which appears to rely on special interactions between proteins and EPA-containing phospholipids [37]. In *Shewanella livingstonensis,* EPA is required for normal membrane organization and cell division at temperatures of around 4 °C but not at 18 °C [38]. Thus, the observed increase of EPA percentage during low-temperature ALE may be partially due to the need to enable the correct expression and folding of membrane proteins under these conditions. By contrast, an increase of the saturated fatty acid proportion was observed under high-salinity stress (Figs. 1b, 2a, b), and the adjustment of membrane permeability needed to avoid an extensive influx of Na^+^ and Cl^−^ ions might explain this response [39]. This trend was also observed in the cells of *Dunaliella* and *Chlamydomonas* spp. [40, 41]. Thankfully, lipid accumulation was increased in ALE-HS30 and ALE-TF30 (Table 1), probably due to the need for storing energy-rich compounds to be able to survive in a high-salinity environment [42]. However, further research is needed to elucidate the mechanisms of lipid upregulation under high salinity.

Lipid synthesis can be induced by various stresses related to nutrient availability and environmental factors, among which nitrogen limitation is recognized as the most successful induction strategy, and is most widely used [3]. However, microorganisms tend to accumulate ROS under these conditions [43]. Moreover, a large number of studies have reported that a higher oxygen supply was beneficial for cell growth and lipid accumulation during the cultivation process of *Schizochytrium* sp. [44]. Despite the fact that over 90% of the oxygen consumed by living organisms is used to produce energy for cell growth and lipid accumulation, there always is an inevitable aspect of its conversion into ROS [45]. At the first stage of fermentation, lower levels of ROS did not cause damage to the cells, and there was a sharp increase of CDW and lipids in both strains during this phase (Fig. 4b). Although it has been documented that nitrogen starvation can induce oxidative stress [43], this was not observed in this study. As shown in Fig. 4a, nitrogen (MSG) was depleted at 36 h, but ROS reached their highest value at 12 h and then gradually declined until 60 h (Fig. 5a). One reason for the apparent peak in ROS levels at 12 h might be the stress experienced by the cells when they were shifted from the nutrient-depleted seed culture medium into a nutrient-rich fermentation medium, and this phenomenon was also observed in other microorganisms [43]. The stage spanning 36–60 h coincides with the exponential growth phase of *Schizochytrium* sp., during which the cells exhibited strong antioxidant enzyme activity which enabled them to resist the increase of ROS levels induced by nitrogen starvation. However, with the increase of cell concentrations in stage II, growth inhibition and nutritional limitation caused high levels of ROS (Fig. 4a) concomitant with a decrease of cell growth and lipid accumulation. In addition, lipid peroxidation can also lead to the accumulation of high levels of ROS [7]. Johansson et al. established a bio-sustainable PUFA production system in *Saccharomyces cerevisiae*, and they found that the PUFA-producing strain accumulated high levels of ROS, which resulted in low viability [46]. The sharp increase of ROS in stage II is therefore partly caused by inevitable lipid peroxidation. Furthermore, Fig. 5a, c also show a synchronous trend between the changes of ROS and MDA, a trend that suggests that oxidative stress is a consequence of an increase of lipid peroxidation (indicated by MDA) brought about by ROS.

*Schizochytrium* sp. only possess one intact pathway containing anaerobic polyketide synthase (PKS) for DHA synthesis, which is distinct from the fatty acid synthase pathway (FAS) [47]. While oxygen limitation strategies lead to an increase of the DHA percentage, they also significantly reduce the biomass productivity, thereby resulting in a decrease of total production and overall productivity. For instance, Jakobsen et al. found that the content of DHA reached 52% in oxygen limited cells, but CDW decreased to 25–27 from 90 to 100 g/L in *Aurantiochytrium* sp. [44]. In addition to oxygen, various stress conditions or lipid peroxidation can also induce ROS generation. However, *Schizochytrium* sp. has a weak ability to remove ROS, which was evidenced by the T-AOC of the parental strain, which decreased to non-detectable levels at 60 h and remained there until the end of fermentation (Fig. 5b). The resulting lipid peroxidation can cause a loss of the produced lipids, which might explain the decrease of the DHA and PUFA percentages in the parental strain after 84 h (Fig. 4c, d). Although the PUFA content of ALE-TF30 increased less and even decreased after 84 h, DHA is more recalcitrant to oxidation than DPA because the Δ4 double bond must first be removed by peroxisomal β-oxidation [48]. Thus, an apparent selective retention of DHA resulted in a continuous increase of the percentage of DHA in TFAs until the end of fermentation. As a consequence, maintenance of ROS detoxification is paramount to the survival of all aerobic life forms. In recent years, an increasing amount of work was done on strategies to scavenge ROS or enhance T-AOC by adding antioxidants to improve microalgal performance. For example, DHA productivity was increased by 20 and 44% in *Crypthecodinium cohnii* and *Schizochytrium* sp. by adding sesamol and ascorbic acid, respectively [9, 49]. In addition, Gaffney et al. improved the T-AOC of *Schizochytrium* sp. via the supplementation of flaxseed oil [50]. Moreover, increasing experimental evidence seems to point in the direction that intracellular ROS may in fact be general mediators of lipid accumulation in oleaginous microorganisms [51]. In fact, many researchers attributed the stress-mediated induction of lipid accumulation to the generation of ROS [52]. Kang et al. used oxidative stress to induce lipid production in *Chlorella vulgaris* [53]. Similarly, Yilanciooglu et al. applied exogenous H_2_O_2_, which resulted in oxidative stress and increased the cellular lipid content up to 44% [54]. However, only a few potential mechanisms of ROS-mediated lipid accumulation have been discovered, and direct experimental evidence is still lacking.

In addition to ROS, microalgae have also evolved adaptive ways to combat salt stress, adjustment of the expression of antioxidant enzymes being one such adaptive mechanism, which is potentially more attractive due to its impact on lipid production. Tang et al. reported that enhanced SOD and CAT activity provides protection to *Scytonema javanicum* by scavenging ROS, thereby mitigating the oxidative burst caused by NaCl stress [55]. Likewise, exposing cells to high doses of NaCl led to a significant rise in the activities of SOD, POD, CAT, and GST in *Nostoc muscorum* and *Phormidium foveolarum* [56]. In the present study, the most striking observation was the overall increase of T-AOC in the ALE-TF30 strain (Fig. 5b). To further explore these results, the gene expression of SOD, CAT, and APX was measured at 60 and 108 h. Oxidative stress generally involves increases in the production of O_2_^−^ and H_2_O_2_. In the evolved strain, the gene expression of SOD and CAT was sharply increased, whereas APX showed only 1.11 and 38% increases over the parent strain at 60 and 108 h, respectively, which indicated that the high T-AOC in ALE-TF30 was mostly attributable to SOD and CAT. In contrast to APX, SOD and CAT act as the first line of defense against oxidative damage, and are crucial for the detoxification of O_2_^−^ and H_2_O_2_ [57], which might explain the lower increase of gene expression of APX in the evolved strain. During the later stages of fermentation, high ROS levels due to nitrogen limitation, growth inhibition, and strong lipid peroxidation may have caused damage to proteins, resulting in lower gene expression or loss of function of antioxidant enzymes. This may be the reason why the genes encoding fatty acid biosynthesis enzymes and antioxidant enzymes were all downregulated in the two strains during the oxidative damage stage (108 h; Fig. 6). This phenomenon was also observed in other microorganisms [55].

The ultimate aim of this study was to develop a strain with high DHA production capacity, which is why key genes associated with fatty acid biosynthesis—FAS, ORFA, ORFB, and ORFC—were also analyzed. The expression of PUFA synthases (ORFA/B/C) was remarkably increased in the evolved strain, which was in agreement with other studies on the effects of low-temperature stress [58]. However, the dramatic rise in the expression of PUFA-related genes resulted in only a moderate increase of PUFA production in the ALE-TF30 strain, which might indicate that the overexpression of polyketide synthases outpaced the cells’ supply of energy or precursors for PUFA synthesis. This was in agreement with many studies which showed that genes related to lipid biosynthesis, such as acetyl-CoA carboxylase and acyl-CoA synthetase, were significant upregulated under conditions of environmental stress [59, 60]. Among the three genes encoding PKS enzymes, ORFC expression was increased the most, and was 4.12- and 3.34-fold higher than that of the parental strain at 60 and 180 h, respectively. ORFC contains two DH (dehydrase) domains and one ER (enoyl-ACP reductase) domain, which showed higher sensitivity to the changes of environmental factors [61]. DH catalyzes the conversion of ketoacyl-ACP to enoyl-ACP, and the ER domain catalyzes the final step of fatty acid chain extension in which it reduces the double bond. The omission of enoyl-ACP reduction determines the formation of the double bonds of PUFAs. Inspired by this, Cheng et al. developed a method for screening *Aurantiochytrium* sp. strains with high DHA yield using inhibitors of enoyl-ACP reductase and synergistic usage of cold stress at 4 °C [62]. The expression level of ORFC might thus explain the changes of fatty acid composition during DHA fermentation.

## Conclusions

To our best knowledge, this is the first comprehensive study to take into account both lipid overproduction and lipid peroxidation in the fermentation process. We explored the cooperative effect of low temperature and high salinity in ALE on the growth and DHA production of *Schizochytrium* sp. The best obtained strain, ALE-TF30, displayed both a good DHA synthesis ability and strong antioxidant capacity. The maximum DHA yield with minimum lipid peroxidation was obtained by the endpoint strain of the evolution process. These major performance changes were accompanied by an overall increase of gene expression levels of antioxidant enzymes and polyketide synthases. Such knowledge provides a new method that can be used in other oil-producing microalgae, and proves that ALE is an effective strategy to generate high-performing strains of *Schizochytrium* sp. for industrial applications.

## Methods

### Microorganism and culture conditions

*Schizochytrium* sp. HX-308 (CCTCC M 209059), isolated from seawater and stored in the China Center for Type Culture Collection (CCTCC), was used in this study. The strain was preserved in 20% (v/v) glycerol at − 80 °C.

The seed culture medium and conditions of propagation were the same as those used in our previous study [63]. After three generations, the seed cultures (1% v/v) were transferred to 500-mL shake flasks containing 100 mL of medium and cultured at 30 °C under constant orbital shaking at 170 rpm, and the resulting cell suspension was used as inoculant for the fermentation. The fermentation medium was the same as reported in our previous study [47]. The basic medium contained 50 g/L glucose and 0.4 g/L yeast extract, which were dissolved in artificial sea water. The artificial sea water contained (g/L): Na_2_SO_4_ 10, (NH_4_)_2_SO_4_ 0.8, KH_2_PO_4_ 4, KCl 0.2, MgSO_4_ 2, monosodium glutamate 20, CaCl_2_ 0.1, and the trace elements solution (g/L): Na_2_EDTA 6, FeSO_4_ 0.29, MnCl_2_·4H_2_O 0.86, ZnSO_4_ 0.8, CoCl_2_·6H_2_O 0.01, Na_2_MoO_4_·2H_2_O 0.01, NiSO4·6H_2_O 0.06, and CuSO_4_·5H_2_O 0.6.

### Adaptive laboratory evolution experiments

**Fig. 7:**
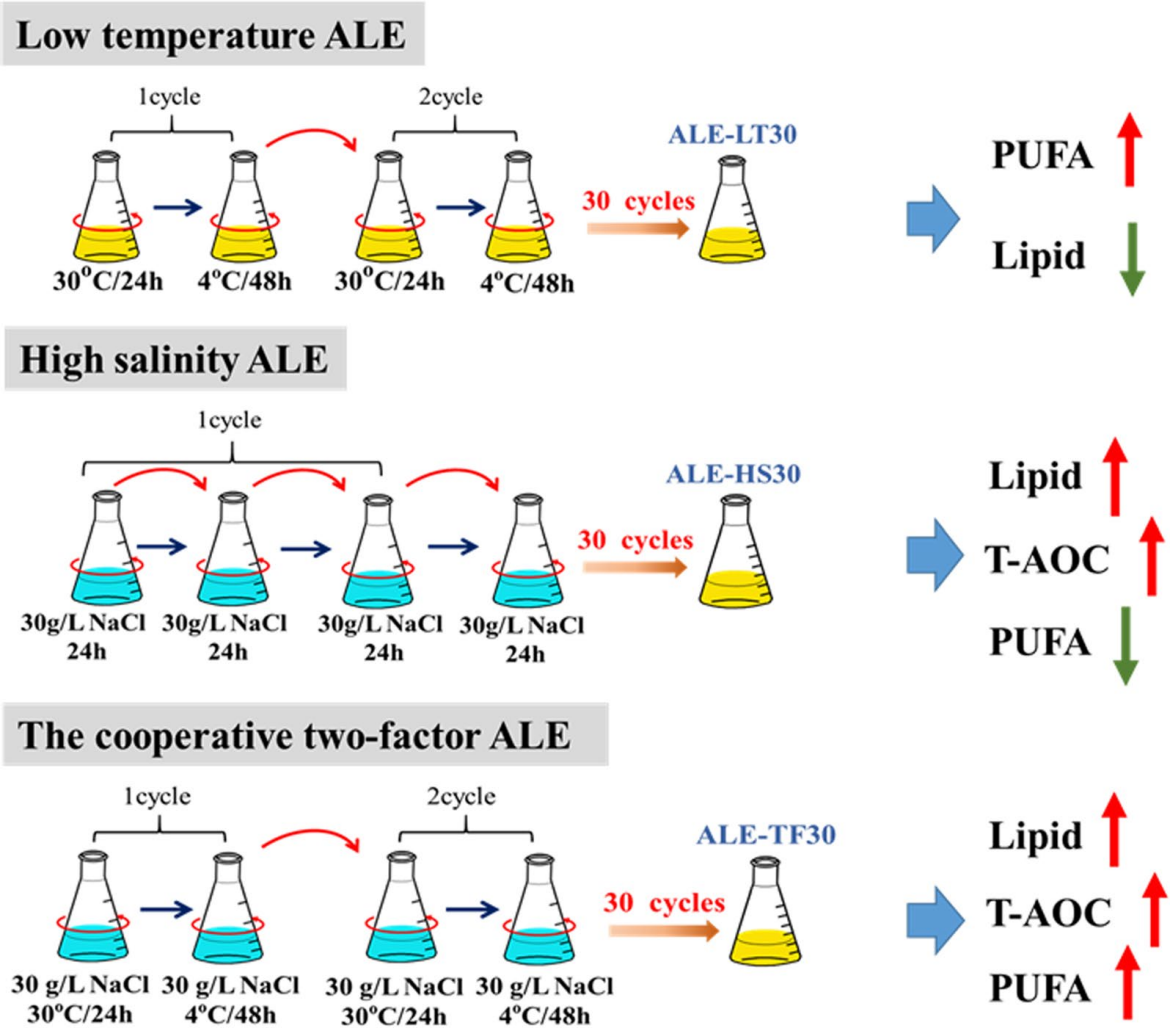
Schematic diagram of the experimental process of the two different adaptive laboratory evolution (ALE) strategies. Yellow represents normal medium and blue represents high-salinity medium

ALE was based on a long-term serial transfer procedure using 4 °C and 30 g/L NaCl as low-temperature and high-salinity stress inducers, respectively. The experimental process was conducted as shown in Fig. 7. For each new cycle, the seed culture (1% v/v) was transferred to fresh medium. In addition to the stress inducer, the culture medium or condition of the evolved strain was the same as for the ancestral strain. Every 24 h, the seed culture (1% v/v) was transferred to a fresh medium. The method of cooperative two-factor ALE is similar to low-temperature ALE, the only difference being high-salinity medium. In this study, every 72 h was defined as one cycle, and three ALE experiments were conducted repeatedly for 30 cycles. After 30 cycles, low-temperature ALE, high-salinity ALE, and the cooperative two-factor ALE strains were named ALE-LT30, ALE-HS30, and ALE-TF30, respectively. The ALE-TF30 strain was the endpoint strain. A serial transfer experiment has proved that the endpoint strain ALE-TF30 had a fixed stable improved phenotype. The fermentation medium was the same as in our previous study [47]. All fermentation experiments were performed in triplicate. Such serial transfers were repeated for 30 cycles and 1 mL samples of the evolving population were taken and stored at − 80 °C in 20% glycerol for subsequent analysis every 5 cycles.

### Determination of cell dry weight and total lipids

Cell dry weight was determined gravimetrically by harvesting 10 mL culture aliquots by centrifugation at 4500×*g* for 5 min. The cells were subsequently transferred to a weighed filter paper and dried at 60 °C to constant weight (~ 12 h). The yield of total lipids, extracellular glucose, and glutamate were determined the same way as published in our previous study [63].

### Fatty acid analysis

Fatty acid methyl esters (FAMEs) were prepared from 0.2 g of dried cells and analyzed using a GC-2010 gas chromatography system (Shimadzu, Japan), equipped with a DB-23 capillary column (60 m × 0.22 mm; Agilent, USA) and flame ionization detector (FID). Nitrogen was used as the carrier gas. The injector was maintained at 250 °C with an injection volume of l μL. The column temperature was raised from 100 to 200 °C at 25 °C/min and then increased to 230 °C at 4 °C/min, followed by keeping this temperature for 9 min. The FID detector temperature was set to 280 °C. FAMEs were identified by comparing the retention times with corresponding external authentic reference standards (Sigma, USA). The quantities of individual FAMEs were estimated based on the integrated peak areas on the chromatogram using non-adecanoic acid (C19:0) as the internal standard.

### Determination of total antioxidant capacity

Intracellular total antioxidant capacity (T-AOC) was determined using the T-AOC assay kit (Angle Gene, China) according to the manufacturer’s instructions. Cells were harvested by centrifugation for 5 min and washed with physiological saline in order to remove the excess of ascorbic acid in the medium. After being disrupted using an ultrasonic disrupter for 15 min, 200 μL of crude extract was mixed with 0.2 mM DPPH radical. The mixture was incubated for 30 min at 37 °C and the absorbance at 517 nm was measured. The radical scavenging ability was calculated based on the difference in the measured decrease of absorbance at 517 nm between the blank and the respective sample, whereby a unit of T-AOC was defined as the increase of OD_517_ per mg cell dry weight, per minute, at 37 °C. The value of T-AOC was calculated using the following equation:

$${\text{T-AOC}}\left( {{\text{U/mg}}\;{\text{CDW}}} \right)\, = \,\frac{{\frac{{{\text{Ar}} - {\text{Ac}}}}{0.01\; *\;30}}}{\text{CDW,}}$$

where Ar is the absorbance of the reaction sample (with DPPH solution) and Ac is the absorbance of the control sample (without DPPH solution).

### Determination of intracellular reactive oxygen species

Intracellular ROS levels were determined according to the method by Li et al. which uses the oxidation-sensitive fluorescent indicator 2′,7′-dichlorofluorescein diacetate (DCFH-DA) [64]. This probe is not fluorescent in its original form and can freely cross cell membranes. Inside the living cells, two acetate groups (DA) are removed from the indicator forming DCFH, which is still not fluorescent. In the presence of ROS, DCFH is oxidized to fluorescent 2′,7′-dichlorofluorescein (DCF), which can be measured by fluorometry. DCFH-DA was added at an in-well concentration of 10 Μm to collected cells, and incubated for 30 min at 37 °C. After a full half-hour of exposure to the probe, the excess of indicator in the medium was washed with PBS to make sure that only the intracellular oxidation was measured. Fluorescence intensity was measured using a fluorescence spectrophotometer at an excitation wavelength of 485 nm and an emission wavelength of 530 nm.

### Measurement of lipid peroxidation

The level of lipid peroxidation was determined by measuring malondialdehyde (MDA) equivalents according to the method of Heath et al. [65]. Treated and untreated cells were homogenized in 5% (w/v) TCA and centrifuged at 10,000×*g* for 10 min at 4 °C. The reaction mixture containing thiobarbituric acid-TCA solution and cell extract was boiled for 20 min. The absorbance of the reaction mixture was recorded at 532 and 600 nm. The values of non-specific absorption recorded at 600 nm were subtracted from the values recorded at 532 nm. The content of MDA equivalents was determined using an extinction coefficient of 155 mM/cm.

### Real-time quantitative PCR

Samples of the parental strain and the ALE-TF30 strain were obtained at 60 and 108 h of fermentation, and RNA was isolated using the Rapid fungal RNA extraction kit (Zoonbio Biotechnology, Nanjing, China). cDNA was obtained using the TURE script cDNA Synthesis Kit (Zoonbio Biotechnology, Nanjing, China) and used for quantitative PCR analysis. Seven target genes (superoxide dismutase, catalase, ascorbate peroxidase, FAS, ORFA, ORFB, and ORFC) were tested in the present study. The relative levels of the amplified mRNAs were evaluated according to the 2^−△△Ct^ method using β-actin for normalization.

## Additional file

**Additional file 1: Table S1.** Endpoint strain of ALE-TF30 was cultured and passaged to sixteen generations at 170 rpm and 30 °C.

## Abbreviations

ALE: adaptive laboratory evolution
APX: ascorbate peroxidase
CAT: catalase
CDW: cell dry weight
DHA: docosahexaenoic acid
DPA: docosapentaenoic acid
EPA: eicosapentaenoic acid
GSH: glutathione
PUFA: polyunsaturated fatty acid
ROS: reactive oxygen species
T-AOC: total antioxidant capacity
TAG: triacylglycerol
TFAs: total fatty acids
TLs: total lipids
MDA: malondialdehyde
SFA: saturated fatty acid
SOD: superoxide dismutase
