# Supplementary material for: Development of a cooperative two-factor adaptive-evolution method to enhance lipid production and prevent lipid peroxidation in Schizochytrium sp
Source: Biotechnol Biofuels. 2018 Mar 14;11:65. doi: 10.1186/s13068-018-1065-4 (PMC5851066; doi:10.1186/s13068-018-1065-4)
Supplement: Supplementary file 1 — Additional file 1: Table S1. Endpoint strain of ALE-TF30 was cultured and passaged to sixteen generations at 170 rpm and 30 °C. [file 13068_2018_1065_MOESM1_ESM.docx]

Table S1. Endpoint strain of ALE-TF30 was cultured and passaged to sixteen generations at 170rpm and 30°C. Data represent the mean values and standard deviations of three replicates for each measurement.

| Endpoint strain  generations | Cell dry weight  (g/L) | DHA percentage in TFAs  (%) |
| --- | --- | --- |
| 0 | 36.2±1.1 | 51.78±0.4 |
| 2 | 37.8±0.3 | 52.23±0.3 |
| 4 | 36.8±0.6 | 54.78±0.7 |
| 6 | 36.3±1.0 | 53.13±0.3 |
| 8 | 35.1±1.2 | 52.76±0.7 |
| 10 | 36.7±1.2 | 53.72±0.4 |
| 12 | 35.2±0.9 | 54.82±0.8 |
| 14 | 38.1±1.7 | 53.39±0.2 |
| 16 | 36.9±0.8 | 52.94±0.5 |
